# Age-Dependent Pleiotropy Between General Cognitive Function and Major Psychiatric Disorders

**DOI:** 10.1016/j.biopsych.2015.08.033

**Published:** 2016-08-15

**Authors:** W. David Hill, Gail Davies, The CHARGE Cognitive Working Group, David C. Liewald, Andrew M. McIntosh, Ian J. Deary

**Affiliations:** a Centre for Cognitive Ageing and Cognitive Epidemiology, University of Edinburgh, Edinburgh, United Kingdom; b Departments of Psychology, University of Edinburgh, Edinburgh, United Kingdom; c Psychiatry, University of Edinburgh, Edinburgh, United Kingdom

**Keywords:** Aging, Autism, Cognitive function, Genetics, Pleiotropy, Schizophrenia

## Abstract

**Background:**

General cognitive function predicts psychiatric illness across the life course. This study examines the role of pleiotropy in explaining the link between cognitive function and psychiatric disorder.

**Methods:**

We used two large genome-wide association study data sets on cognitive function—one from older age, *n* = 53,949, and one from childhood, *n* = 12,441. We also used genome-wide association study data on educational attainment, *n* = 95,427, to examine the validity of its use as a proxy phenotype for cognitive function. Using a new method, linkage disequilibrium regression, we derived genetic correlations, free from the confounding of clinical state between psychiatric illness and cognitive function.

**Results:**

We found a genetic correlation of .711 (*p* = 2.26e-12) across the life course for general cognitive function. We also showed a positive genetic correlation between autism spectrum disorder and cognitive function in childhood (*r*_g_ = .360, *p* = .0009) and for educational attainment (*r*_g_ = .322, *p* = 1.37e-5) but not in older age. In schizophrenia, we found a negative genetic correlation between older age cognitive function (*r*_g_ = −.231, *p* = 3.81e-12) but not in childhood or for educational attainment. For Alzheimer’s disease, we found negative genetic correlations with childhood cognitive function (*r*_g_ = −.341, *p* = .001), educational attainment (*r*_g_ = −.324, *p* = 1.15e-5), and with older age cognitive function (*r*_g_ = −.324, *p* = 1.78e-5).

**Conclusions:**

The pleiotropy exhibited between cognitive function and psychiatric disorders changed across the life course. These age-dependent associations might explain why negative selection has not removed variants causally associated with autism spectrum disorder or schizophrenia.

General cognitive function describes the positive correlations that exist between seemingly disparate tests of cognitive function (1). This overlap accounts for around 40% of the variation in cognitive test scores (2). Individual differences in cognitive function are predictive of both physical and mental health (3). Ageing has been shown to have differential effects on the facets of cognitive function, whereby tests that include novel information and/or invoke on-the-spot abstract reasoning skills, often termed tests of general fluid cognitive function, show decline from early adulthood onward (4, 5). Tests of crystallized function that include measurements of learned material, such as vocabulary, are more robust to the ageing process (6).

Higher general cognitive function in childhood is predictive of lower self-reported psychological distress decades later (7). This association between cognitive function and psychological well-being extends to severe psychiatric conditions, with a 1 standard deviation lower score in general cognitive function being associated with a 60% increased risk for hospitalization for schizophrenia, a 50% increase for mood disorders, and a 75% greater risk for alcohol-related disorders in two decades of follow-up (8). Indeed, a higher risk of several psychiatric conditions has been associated with a lower level of cognitive function, including major depressive disorder (9, 10), attention-deficit/hyperactivity disorder (ADHD) (11), and autism spectrum disorder (ASD). There is also evidence for lower premorbid cognitive function in individuals who later develop bipolar disorder (12, 13), although higher premorbid cognitive function may also confer risk (14).

Using genome-wide association study data, it has been shown that general cognitive function is a highly polygenic trait influenced by many common genetic variants (single nucleotide polymorphisms [SNPs]) that each exert only a very small effect on phenotypic variation (15). The genome-wide complex trait analysis method of estimating SNP-based heritability finds that common genetic variants tag causal variants that together account for around 30% of general cognitive ability variation (15, 16, 17, 18, 19). However, even in a meta-analysis of over 53,000 subjects, the variance accounted for by genome-wide significant loci was only a small fraction of this figure (15).

The genetic architecture of psychiatric disorders is also highly polygenic, whereby the combined effect of many common variants accounts for much of the heritability, and as with cognitive function, the number of genome-wide significant loci do not explain the heritability accounted for by all SNPs (20). The present study tests the hypothesis that the phenotypic link between general cognitive function and psychiatric disease is, at least in part, caused by pleiotropic genetic variants.

Pleiotropy between traits can be inferred by applying the polygenic prediction method to genome-wide association study (GWAS) data sets (21). This method, often termed polygenic risk scoring, uses markers selected from the summary GWAS data in one sample to construct a weighted genetic risk score for each individual in an independent sample. An association between this composite score and the trait of interest in the second sample is evidence of an overlap between the polygenic architecture of each trait. This method has been used to indicate that there are shared genetic influences between, for example, higher risk of schizophrenia and lower cognitive function in older age and more age-related cognitive decline (22, 23) and between a greater risk of ASD and higher cognitive function (24). However, the polygenic prediction method suffers from a number of limitations, including requiring access to genotype data, as well as producing biased results in cases where the discovery and replication samples overlap (25, 26). By placing these additional constraints on the type of samples that can be used, the total number of available participants, along with statistical power, falls. In addition, there is a further loss of power as informative markers may be lost due to pruning for linkage disequilibrium (LD) (27).

Another method to quantify a genetic correlation is restricted maximum likelihood analysis (28), where genotype data are from the same group of individuals assessed on multiple traits (29) or across multiple time points (30). This method allows the full number of genome-wide markers to be included in the analysis, meaning that, by contrast with the use of polygenic risk scores, data are not lost through pruning. However, this method suffers from the limitation that the same individuals are required to have been assessed on multiple traits. While this, at best, presents logistical difficulties in the recruitment of a sample, in the case of psychiatric disorders, the symptoms displayed by diagnosed individuals may interfere with the cognitive test process resulting in an inflation of the genetic correlation.

Here, to avoid the limitations associated with bivariate restricted maximum likelihood and polygenic prediction, we used a new method, LD regression (25, 31), to derive a genetic correlation using GWAS summary data. LD regression exploits the finding that SNPs in regions of high LD provide a measure of a greater proportion of the genome than SNPs in regions of low LD. This means that, assuming a polygenic architecture, SNPs in regions of high LD are more likely to capture variance associated with a causal variant, leading to an increase in the association test statistics from a GWAS proportional to the level of LD in the region (32). As consequence of this, LD can be used to predict GWAS test statistics. This logic extends to the bivariate design where, at each loci, the product of the test statistics from two GWAS replaces the association statistics from a single GWAS. In the presence of a nonzero genetic correlation between the traits measured by the two GWAS, the level of LD can be used to predict the product of association statistics at each loci (25, 31).

We used LD regression, first used by Bulik-Sullivan *et al.* (25, 31) to derive 300 genetic correlations between 25 traits. Here, we focused on a smaller number of traits to provide detailed analyses of pleiotropy between cognitive abilities and psychiatric disorders and tests of whether these associations change over time. In addition, we assessed the validity of the use of educational attainment as a proxy phenotype for cognitive function in genetic correlations with psychiatric disorders. In the current study, we derived genetic correlations between general cognitive function in the 53,949 individuals of the Cohorts for Heart and Aging Research in Genomic Epidemiology (CHARGE) consortium (age range 45–102) (15) and the seven major psychiatric disorders. The disorders examined were schizophrenia (33), bipolar disorder (34), major depressive disorder (MDD) (35), ASD (20), attention-deficit/hyperactive disorder (20), anorexia nervosa (36), and Alzheimer’s disease (37). In addition, we used the summary data from the GWAS meta-analysis of the Childhood Intelligence Consortium (CHIC), which examined cognitive function in childhood (*n* = 12,441, age range 6–18) (17). We also used summary data from a GWAS meta-analysis of educational attainment (38), defined as whether or not a college level of education was achieved. Only one educational attainment variable was used from this article by Rietveld *et al.* (38), as they have been previously shown to genetically correlate at *r*_g_ = 1.0 (25). The assessment of cognitive function in both older age and childhood allows for a genetic correlation for cognitive function to be derived across these two time points; it also allows for a more mechanistic interpretation of any cognitive function-psychiatric disorder genetic correlation that is found, as variants responsible for the development of cognitive function (childhood general cognitive function) and variants that exert a protective effect against the ageing process (older age general fluid cognitive function) may exert differential risk/protective effects against psychiatric disease. We also included educational attainment to examine its use as a proxy phenotype for general cognitive function.

## Methods and Materials

### Samples

Summary data from GWAS meta-analyses of schizophrenia, bipolar disorder, MDD, ADHD, ASD, anorexia nervosa, Alzheimer’s disease, older age general fluid cognitive function, childhood general cognitive function, and educational attainment were used to derive genetic correlations. Table 1 provides details pertaining to the reference and the source of the data for each of the phenotypes examined here.

### Statistical Analysis

We implemented the same data processing pipeline as implemented by Bulik-Sulivan *et al.* (25). First, a heritability *Z* score of >4 and a mean χ^2^ statistic of >1.02 were used as cutoffs to ensure that the GWAS data sets used contained a sufficiently clear polygenic signal contributing toward heritability. As the ADHD data set failed both of these quality control procedures, it was removed from further analyses (Table 2). Minor allele frequency (MAF) was filtered to include SNPs where MAF > .01. To ensure high imputation quality, SNPs were filtered to HapMap3 (https://www.sanger.ac.uk/resources/downloads/human/hapmap3.html) SNPs with 1000 Genomes European MAF > .05 (http://www.genomicsengland.co.uk/the-100000-genomes-project/). Indels and structural variants were removed along with strand-ambiguous SNPs. Additionally, SNPs whose alleles did not match those in the 1000 Genomes were also removed. Finally, as outliers can increase the standard error in a regression, genome-wide significant SNPs were removed, as were SNPs with very large effect sizes (χ^2^ > 80). As the GWAS used here were of European ancestry, the precomputed LD scores and weights were taken from the Broad Institute (Cambridge, Massachusetts; http://www.broadinstitute.org/∽bulik/eur_ldscores/). As it was not possible to quantify the degree to which these samples overlapped, the intercept of the regression model describing the genetic correlation between the pairs of traits was left unconstrained. Differences between the genetic correlations produced by LD regression were explored by deriving a *Z* score for the difference between the two. Additionally, in the case of the Alzheimer’s disease phenotype, a region encompassing 500 kilobase around each side of the *APOE* locus was removed to ensure any genetic correlations derived were not being driven solely by the large effects found within this region.

## Results

Univariate heritability analysis was performed on each of the consortia’s phenotypes using LD regression (25). These results are reported in Table 2. It should be noted that due to the implementation of genomic control factors to control for population stratification, these heritability estimates will be biased downward.

There was a significant genetic correlation between general fluid cognitive function in older age and general cognitive function in childhood, *r*_g_ = .711, SE = .101, *p* = 2.256e-12, indicating substantial pleiotropy across the human life course. Educational attainment showed a high and significant genetic correlation with childhood general cognitive function, *r*_g_ = .719, SE = .089, *p* = 8.137e-16, demonstrating a high level of pleiotropy between these two traits. The genetic correlation between older age cognitive function and educational attainment was not computed because permission was not granted by the CHARGE consortium to perform these analyses. We next examined these ages separately to discover if the polygenic variation at each age overlapped with that of the six psychiatric disorders.

Table 3 shows the results of our testing for pleiotropy between cognitive function in older age and in younger age with major psychiatric disorders. These genetic correlations were derived using LD regression (25, 31) along with the publically available data sets specified in Table 1. The polygenic component to childhood general function and educational attainment overlapped substantially and positively with ASD, *r*_g_ = .360 (SE = .108, *p* = .0009) and *r*_g_ = .322 (SE = .074, *p* = 1.37e-5), indicating that both childhood cognitive function and educational attainment demonstrate pleiotropy with ASD. This is consistent with earlier reports where polygenic scores indicated that a greater risk for ASD predicted phenotypic variance for higher cognitive function (24). This finding did not extend to the sample of older age general cognitive function (*r*_g_ = .058, SE = .071, *p* = .413), despite its having greater power than the childhood cognitive function data set. This indicates that the positive genetic overlap between greater ASD risk and higher cognitive function is driven by variants that exert their effects on the development of cognitive function rather than its maintenance in older age. The difference between the genetic correlations of childhood cognitive ability and older age cognitive ability was statistically significant*, Z* = 2.337, *p* = .019, showing that the genetic correlation between childhood general cognitive function was significantly greater than the genetic correlation between ASD and older age cognitive function. None of the genetic correlations between childhood cognitive function and other psychiatric disorders were significant.

There was a significant negative genetic correlation between cognitive function in older age and schizophrenia (*r*_g_ = −.231, SE = .033, *p* = 3.806e-12), i.e., greater genetic risk of schizophrenia was associated with lower cognitive function in that age period. The direction of effect and presence in older age and not childhood (*r*_g_ = −.044, SE = .057, *p* = .443) or in educational attainment (*r*_g_ = .060, SE = .036, *p* = .093) is consistent with findings using polygenic scoring methods (22, 23). Again, the difference between the genetic correlations of older age general fluid cognitive function and childhood general cognitive function with schizophrenia were statistically significant, *Z* = 2.839, *p* = .005. These results indicate that it is the unique genetic elements of older age general fluid cognitive function that are responsible for the pleiotropy with schizophrenia.

For educational attainment, significant genetic correlations were found with bipolar disorder (*r*_g_ = .264, SE = .062, *p* = 2.15e-5), MDD (*r*_g_ = −.144, SE = .082, *p* = 8.06e-2), and anorexia nervosa (*r*_g_ = .171, SE = .051, *p* = 7.14e-4). The positive genetic correlations with bipolar disorder and anorexia nervosa indicate that the genetic variants that contribute toward these disorders also serve to increase educational attainment.

## Discussion

We used the LD regression method, developed by Bulik-Sullivan *et al.* (25, 31), to test genetic correlations (based on common genetic markers) between general cognitive function in childhood and older age, educational attainment, and major psychiatric disorders. We report several novel findings. We found the first significant genetic correlation between cognitive function in childhood and older age. We found a positive genetic correlation between autism spectrum disorder and cognitive function in childhood and in educational attainment but not older age. We found a negative genetic correlation between schizophrenia and cognitive function in older age but not childhood or for educational attainment. We found genetic correlations between Alzheimer’s disease and all three measures of cognitive function across the life course.

The LD regression method does not require the same individuals to be measured in the same phenotype and so the presence of the symptoms associated with these disorders cannot confound the results of this study. In addition, the overlapping control participants did not bias these results as they would have done with polygenic scoring (27), meaning that all genotyped participants could be included, keeping statistical power relatively high.

The genetic correlation between childhood general cognitive function and older age was *r*_g_ = .711, SE = .101, *p* = 2.256e-12, indicating that whereas a significant proportion of the genetic variance was shared between the mean ages of these cohorts (CHIC = 10.96 years, age range 6–18; CHARGE = 66.40 years, age range 45–102), they were not identical and possessed unique genetic factors. The magnitude of this correlation was larger, though similar, to that previously reported using longitudinal samples (30). Phenotypic heterogeneity between the CHIC and the CHARGE consortia may have led to an attenuation of this correlation, as the measure of general cognitive function extracted from different batteries of cognitive tests has been shown to correlate between .79 and 1 (39, 40).

Childhood cognitive function showed a large genetic correlation with educational attainment (*r*_g_ = .719), indicating a substantial level of pleiotropy between these two phenotypes. This finding provides support for the idea that educational attainment can be used as a proxy phenotype (41, 42) for childhood cognitive function, as the same genetic variants are associated with individual differences in both of these variables.

The statistically significant positive overlap between the polygenic components of ASD and childhood general cognitive function builds on the findings of Clarke *et al.* (24), who reported a positive association between the polygenic score derived from a GWAS on ASD that could predict a significant proportion of the variance for general cognitive function in a sample of 52-year-olds (beta = .068, *p* = 6e-7) and in a sample of 17-year-olds (beta = .073, *p* = .029) but not in a sample of 70-year-olds (beta = .02, *p* = .55) or 79-year-olds (beta = −.03, *p* = .48), indicating that as the age of the cohort increased, the degree to which the polygenic architecture of ASD overlapped with cognitive function was attenuated. While the modest sample sizes used by Clarke *et al.* (24) precluded a formal test of this hypothesis, here we incorporate much larger sample sizes in two nonoverlapping age groups to show that, during childhood, the polygenic contribution of common variants to ASD overlaps positively and substantially with common SNPs tagging the variants responsible for childhood general cognitive function, but by older age, pleiotropic common variants between cognitive function and ASD are not found. This indicates that the lack of association between polygenic scores derived using ASD data sets and childhood cognitive ability have been underpowered (24).

The direction of this effect, an increase in the common variants underlying general cognitive function, meaning an increase in common risk alleles to autism, appears to contrast with earlier results using the twin method where negative genetic correlations were found in childhood (43, 44). However, in these studies, both ASD status and general cognitive function were assessed in the same individuals. Should the symptoms of ASD impact on test performance, any alleles truly associated with ASD would also show negative associations with cognitive test scores. The present study disentangles these confounds by measuring cognitive functioning in a separate sample from those with ASD.

A negative genetic correlation between ASD and cognitive function has also been suggested, as de novo copy number variation at 16p11.2, associated with ASD (45), has also been shown to be associated with cognitive function in non-ASD control subjects (46). However, the discrepancy between these previous studies and the current study can be attributed to the frequency of the genetic variants being studied. While rare or de novo variants associated with ASD may have deleterious consequences for cognitive function, the common polygenic architecture of ASD is associated with an increase in cognitive functioning.

The genetic correlation between older age general cognitive function and schizophrenia is consistent with previous reports featuring the polygenic scoring method (22, 23). Another study using polygenic scores assembled using 9394 cases and 12,462 control subjects (47) showed those with greater polygenic scores derived from a schizophrenia data set showed a lower level of cognitive function in older age, as well as a greater level of cognitive decline from age 11 to age 70 (22). However, in the same study, no such effect was found using individuals at age 11. The finding that the polygenic component associated with schizophrenia is associated with only older age general cognitive function indicates that it is the variants that are protective of age-related cognitive decline, rather than the variants responsible for the development of cognitive function or the development of cognitive function, that drive the genetic correlation between cognitive function and schizophrenia.

The positive genetic correlation between educational attainment and ASD, as well as the lack of genetic correlation between educational attainment and schizophrenia, indicates that whereas educational attainment can serve as a proxy phenotype for childhood cognitive ability, it does not capture the same genetic variants as older age cognitive function. The data sets on educational attainment assembled by Rietvald *et al.* (38) were composed of individuals who were aged 30 years and below, and as the polygenic burden for schizophrenia acts later in life to increase the rate of cognitive decline (22), it may be that these participants were too young to experience the results of any polygenic effects that might manifest later in the life course.

Educational attainment also demonstrated a positive genetic correlation between bipolar disorder and with anorexia nervosa, as first reported by Bulik-Sullivan *et al.* (25). The results for bipolar disorder follow the same trend as childhood cognitive ability but were not significant in the smaller childhood sample. Previous work examining educational attainment and the risk of bipolar disorder has shown that risk increases at both ends of the educational attainment spectrum, with children attaining very high grades showing the greatest degree of risk (48). The results of the current study provide molecular genetic evidence that high cognitive function before age 30 is influenced by some of the genetic variants that influence the onset of bipolar disorder. The lack of association with childhood ability in the current study may indicate a lack of power in the sample. For anorexia nervosa, the positive genetic correlation with educational attainment concurs with some reports indicating that those with anorexia nervosa score higher on tests of cognitive function (49). The failure to find a genetic correlation in either the older or younger samples on general cognitive function may indicate a lack of power or age-dependent pleiotropy. The genetic correlation with older age cognitive function was .11 (nominally significant but did not survive correction for multiple testing) and was not very different from that of .17 for educational attainment. The lack of genetic correlation between schizophrenia and educational attainment indicates that whereas educational attainment could be used as a proxy phenotype for cognitive function in childhood, it does not capture the relevant genetic variants involved in cognitive function in older age.

The notion of an age-dependent association between common variants and cognitive function has been previously demonstrated with rs10119 located in the *APOE*/*TOMM40* region (15). It was shown that between the ages of 55 and 80, there is a significant negative correlation between the effect size of the A allele of the rs10119 locus and age, and at age 55, the effect for this allele was 0. The average age of the older age cohort here was 66.4 years, meaning that they were an age where age-dependent genetic effects on cognitive function are known to come into play. Here, we provide the first molecular genetic evidence that it is the combined signal from these alleles that exhibit pleiotropy with schizophrenia.

Alzheimer’s disease demonstrated similar pleiotropy with each of the three cognitive measures used here: childhood cognitive function (*r*_g_ = −.341), older age cognitive function (*r*_g_ = −.324), and educational attainment (*r*_g_ = −.324). This indicates that across the life course, variants influencing Alzheimer’s disease contribute toward cognitive function. This association was not driven by the *APOE* locus, as this region was removed from the analysis. This finding provides molecular genetic evidence for the system integrity hypothesis, which states that high levels of cognitive function are, in part, the product of a body that has complex systems that are better integrated, which, in turn, makes it resistant to disease (50). As this association begins in childhood and is of the same magnitude in older age, it does not appear to be driven by age-related decline but rather indicates that the genotypic blueprint that is resistant to Alzheimer’s disease is also associated with higher levels of the stable trait of general cognitive function through much of the life course.

The age-dependent links with cognitive function found here can help explain why such risk variants remain common in the population. As cognitive function is associated with indicators of fitness including mortality (51) and health (3), this antagonistic pleiotropy between ASD and childhood general cognitive function provides, perhaps, some of the explanation why negative selection has not removed deleterious variants from the population. In the case of schizophrenia, providing the environmental antecedents of schizophrenia are absent, such variants appear to be deleterious to cognitive function in later life. This could indicate that such variants can survive in a population as they are passed to the next generation before the harmful effects are made apparent.

The results of this study provide evidence that there is a substantial overlap between the genetic variants that affect cognitive function across the life span. In addition, we show that the unique genetic variants responsible for cognitive variation in older age overlap with the polygenic component of schizophrenia and in the case of childhood cognitive function ASD. These findings add to the body of knowledge detailing the pleiotropy between normal cognitive variation and mental illness.

## Figures and Tables

**Table 1:** The Samples Used With References

Phenotype	Sample Size	Reference	Data
Older Age General Fluid Cognitive Function	53,949	Davies *et al.*, *Molecular Psychiatry* (2015) (15)	N/A
Childhood General Cognitive Function	12,441	Benyamin *et al.*, *Molecular Psychiatry* (2013) (17)	http://ssgac.org/documents/CHIC_Summary_Benyamin2014.txt.gz
Educational Attainment	95,427	Rietveld *et al.*, *Science* (2013) (38)	http://ssgac.org/documents/SSGAC_Rietveld2013.zip
Schizophrenia	70,100	Schizophrenia Working Group of the Psychiatric Genomics Consortium, *Nature* (2014) (33)	http://www.med.unc.edu/pgc/files/resultfiles/pgc.scz.2012-04.zip
Alzheimer’s Disease	54,162	Lambert *et al.*, *Nature Genetics* (2013) (37)	http://www.pasteur-lille.fr/en/recherche/u744/igap/igap_download.php
Bipolar Disorder	16,731	Psychiatric GWAS Consortium Bipolar Disorder Working Group, *Nature Genetics* (2011) (34)	http://www.med.unc.edu/pgc/files/resultfiles/pgc.bip.2012-04.zip
MDD	18,759	Major Depressive Disorder Working Group of the Psychiatric GWAS Consortium *et al.*, *Molecular Psychiatry* (2013) (35)	http://www.med.unc.edu/pgc/files/resultfiles/pgc.mdd.2012-04.zip
ADHD	5422	Neale *et al.*, *Journal of American Academy of Child and Adolescent Psychiatry* (2010) (20)	http://www.med.unc.edu/pgc/files/resultfiles/pgc.adhd.2012-10.zip
ASD	10,263	Cross-Disorder Group of the Psychiatric Genomics Consortium, *Lancet* (2013) (20)	http://www.med.unc.edu/pgc/files/resultfiles/pgc.cross.aut.zip
Anorexia Nervosa	17,767	Boraska *et al.*, *Molecular Psychiatry* (2014) (36)	http://www.med.unc.edu/pgc/files/resultfiles/gcan_meta-out.gz

The final column, Data, contains links to the data sets used. The data set pertaining to older age cognitive function is not publically available.

Educational attainment indicates if a college or university degree was obtained. A sample size of 26,474 was used to derive the heritability of older age general fluid cognitive function in the CHARGE cohort as the quality control procedures used included an SNP if it was present in at least half of the cohort.

ADHD, attention-deficit/hyperactivity disorder; ASD, autism spectrum disorder; CHARGE, Cohorts for Heart and Aging Research in Genomic Epidemiology; MDD, major depressive disorder; SNP, single nucleotide polymorphism.

**Table 2:** Results of the Quality Control Procedures Implemented to Test the Suitability of Each Data Set for Use With LD Regression

Reference Phenotype	Target Phenotype	Observed Heritability	Observed Heritability SE	Heritability *Z* Score	Mean χ^2^
Childhood General Cognitive Function	Older age general fluid function	.304	.031	9.806	1.170
Educational attainment	.719	.089	8.079	1.244
Schizophrenia	.526	.025	21.040	1.861
Bipolar disorder	.467	.045	10.378	1.190
MDD	.157	.032	4.906	1.079
ADHD	.188	.102	1.843	1.018
ASD	.419	.059	7.102	1.073
Anorexia nervosa	.315	.032	9.844	1.085
Alzheimer’s disease	.070	.012	5.785	1.107
Older Age General Fluid Cognitive Function	Childhood general cognitive function	.274	.047	5.830	1.076
Schizophrenia	.529	.023	23.000	1.800
Bipolar disorder	.456	.043	10.604	1.186
MDD	.152	.031	4.903	1.077
ADHD	.245	.101	2.426	1.017
ASD	.459	.060	7.650	1.066
Anorexia nervosa	.503	.028	17.964	1.058
Alzheimer’s disease	.062	.011	5.636	1.103
Educational Attainment	Childhood general cognitive function	.272	.047	5.787	1.076
	Schizophrenia	.524	.027	19.407	1.815
	Bipolar disorder	.462	.045	10.267	1.187
	MDD	.156	.031	5.032	1.077
	ADHD	.173	.103	1.680	1.019
	ASD	.443	.060	7.383	1.069
	Anorexia nervosa	.422	.031	13.613	1.064
	Alzheimer’s disease	.065	.012	5.417	1.104

The cutoffs used here were a heritability *Z* score of >4 and a mean χ^2^ statistic of >1.02. These figures were derived using SNPs common to both the reference and the target phenotypes and so may fluctuate between comparisons. The metrics of observed heritability, observed heritability SE, heritability *Z* score, and mean χ^2^ columns pertain to the target phenotype. The ADHD cohort showed a lack of power to detect a consistent polygenic signal for use with LD regression when compared with both reference phenotypes. Educational attainment indicates if a college or university degree was obtained.

ADHD, attention-deficit/hyperactivity disorder; ASD, autism spectrum disorder; LD, linkage disequilibrium; MDD, major depressive disorder; SE, standard error; SNP, single nucleotide polymorphism.

**Table 3:** The Genetic Correlations Between Childhood General Cognitive Function, Old Age General Fluid Cognitive Function, Educational Attainment, and Major Psychiatric Disorders

	Childhood General Cognitive Function			Old Age General Fluid Cognitive Function			Educational Attainment		
Trait	*r*_g_	SE	*p* Value	*r*_g_	SE	*p* Value	*r*_g_	SE	*p* Value
Schizophrenia	−.044	.057	.443	−.231	.033	3.81e-12^a^	.060	.036	.093
Bipolar Disorder	.136	.089	.125	−.133	.056	.017	.264	.062	2.15e-5^a^
MDD	.033	.134	.805	−.118	.094	.210	−.144	.082	8.06e-2
ASD	.360	.108	.0009^a^	.058	.071	.413	.322	.074	1.37e-5^a^
Anorexia Nervosa	.060	.102	.560	.109	.044	.013	.171	.051	7.14e-4^a^
Alzheimer’s Disease	−.341	.106	.001^a^	−.324	.075	1.78e-5^a^	−.324	.074	1.15e-5^a^

Educational attainment indicates if a college or university degree was obtained. *p* values displayed are uncorrected.

ASD, autism spectrum disorder; MDD, major depressive disorder; *r*_g_, genetic correlation; SE, standard error.

a Results passed Bonferroni correction (.05/18 = .003).
